# Characterization of Purified Red Cabbage Anthocyanins: Improvement in HPLC Separation and Protective Effect against H_2_O_2_-Induced Oxidative Stress in HepG2 Cells

**DOI:** 10.3390/molecules24010124

**Published:** 2018-12-31

**Authors:** Sheng Fang, Fubin Lin, Daofeng Qu, Xianrui Liang, Liping Wang

**Affiliations:** 1 School of Food Science and Biotechnology, Zhejiang Gongshang University, Hangzhou 310018, China; linfubinwin@126.com (F.L.); daofeng@zjsu.edu.cn (D.Q.); wanglp155fs@sina.com (L.W.); 2 College of Pharmaceutical Sciences, Zhejiang University of Technology, Hangzhou 310014, China; liangxrvicky@zjut.edu.cn

**Keywords:** anthocyanins, HPLC, temperature, intracellular oxidative stress

## Abstract

In this study, the chemical profiles and antioxidant activities of red cabbage anthocyanin (RCA)-enriched extract are evaluated. The effects of column temperature on the HPLC resolution of the RCAs are studied. The HPLC resolutions became better as the column temperature increased from 20 °C–45 °C. An optimized HPLC condition was achieved at 45 °C and used for the quantification and qualification of the RCAs. The anthocyanins in the enriched powder are all derivatives of cyanidin (268 ± 2 μg/mg), mainly with 19% nonacylated, 51% monoacylated, and 31% diacylated structures with ferulic, sinapic, *p*-coumaric, and caffeic acids characterized by HPLC-MS. The RCA extracts markedly reduced intracellular oxidative stress production by H_2_O_2_ on HepG2 cells and consequently ameliorated cell apoptosis and improved viability. The analytical method and cellular antioxidant activity demonstration of the RCAs will greatly facilitate their functional applications.

## 1. Introduction

Currently, there is an increase in the application of natural anthocyanins as an alternative to synthetic pigments [1,2]. Red cabbage (RC) is a good natural resource of anthocyanins since it has high content and productivity compared to berries [3,4]. The red cabbage anthocyanins (RCAs) have a wide range of color spectrum and hues, as well as excellent thermostability because of their highly-acylated structures [4]. The characterization of anthocyanin profiles and antioxidant activities of purified RCAs are important for its utilization as substitutes for synthetic colorants in the food industry [5].

High-performance liquid chromatography (HPLC) is an extensively-used and powerful technique to separate and characterize anthocyanins in plants including RC [3,4,6,7]. However, because of their similar structures, some anthocyanins in RC have a tendency to be co-eluted in an HPLC column [6], which makes it difficult to achieve a baseline separation. Zhang et al. [7] found that, although some peaks were isolated at the baseline after optimization in a reversed-phase HPLC, there were still other predominant RCAs eluted together either in a C18 or phenyl-hexyl column. Methods based on a two-dimensional HPLC for the analysis of RCAs have also been developed [7,8]. To achieve satisfactory peak resolution, most attention has been focused on the optimization of the speed, pH, and composition of the mobile phase, etc. In this study, we found that the column temperature greatly influenced the separation of RCAs in a reversed-phase HPLC. By optimizing the column temperature, a good resolution of RCAs in a normal C18 column could be achieved. To our knowledge, this is the first time that the column temperature has been recognized as an important variable in the HPLC separation of RCAs, which also provides a good reference for anthocyanins’ analysis from other resources.

The antioxidant effect of anthocyanins is one of the underlying mechanisms for their biological activities [9]. The antioxidant activities of RCAs have always been determined by chemical methods such as the DPPH and ABTS assays [10]. Reactive oxygen species (ROS), which have been implicated in the etiology of various chronic diseases, are a series of metabolic byproducts involved in pathological and degenerative processes in the human body [11]. The determination of ROS level in cells can represent their oxidative stress and consequently demonstrate the antioxidant activities of test compounds in protecting cells [12,13,14]. The protective effects of many natural compounds on the H_2_O_2_-induced oxidative stress in different cells have been demonstrated [15,16]. So far, no studies have been undertaken to investigate the potential protective properties of RCAs against oxidative stress induced by H_2_O_2_ on human hepatocellular carcinoma (HepG2) cells [17,18]. Here, we applied a high content analysis (HCA)-based technology [19] to measure simultaneously the effects of RCAs on cell viability and ROS levels of H_2_O_2_-treated HepG2 cells.

In this study, new methods are established to characterize the properties of RCA extracts that are purified by resin adsorption, including the characterization of anthocyanin profiles and antioxidant activity on HepG2 cells. A new HPLC method with a good resolution of each RCAs was developed by optimizing the column temperatures. Based on the optimized HPLC conditions, the anthocyanin profile of the RCAs was quantitatively and qualitatively determined. Finally, the antioxidant activity of the RCAs was characterized by the ROS-reducing effect of HepG2 cells after H_2_O_2_ treatment. The purification, analytical method, and antioxidant activity demonstration of the RCAs extracts will facilitate their applications as substitutes for synthetic colorants in the food industry.

## 2. Results and Discussions

### 2.1. Effect of Temperature on the HPLC Resolution of RCAs

**Figure 1:**
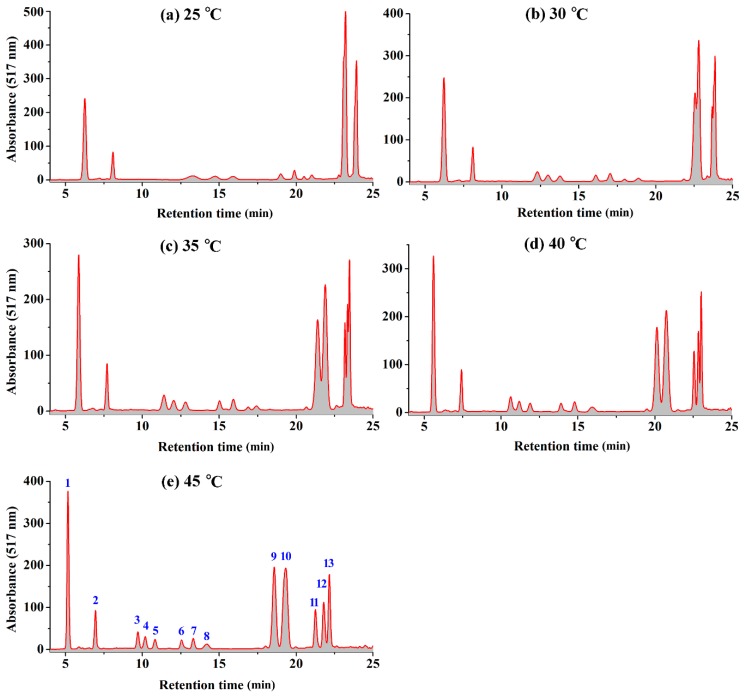
The illustration of the HPLC profiles of the red cabbage anthocyanins at different temperatures: (**a**) 25 °C; (**b**) 30 °C; (**c**) 35 °C; (**d**) 40 °C; (**e**) 45 °C.

An optimum HPLC condition is essential for the qualitative and quantitative analysis of the RCAs extracts. The column temperature is one factor that influences chromatographic retention and selectivity [20], but it is often overlooked in the analytical method of anthocyanins. In this study, an acidic mobile phase with pH 2.0 was optimized to separate RCAs on a reversed phase HPLC-column at 25 °C at first, as shown in Figure 1a. Although the separation in the chromatograph seemed acceptable, there were still some shoulder peaks, as shown in the figure. It is interesting to find that the temperature affects the resolution of RCAs in HPLC remarkably. As shown in Figure 1, the peaks of 9 and 10 and the peaks of 11, 12, and 13 could not be separated at 25 °C. However, as the column temperature increased from 25–45 °C, these peaks became better separated. Generally, as the column temperature increased from 20–45 °C, the retention times of all the RCAs decreased. Consequently, taller and narrower chromatographic peaks could be found in the chromatographic profiles at a higher temperature, as shown in Figure 1e. With mass spectral characterization, for example, the last peak in Figure 1a contained three cyanidin-derivatives with different glycosylation patterns (Peaks 11, 12, and 13 were characterized as cyanidin 3-(feruloyl)(feruloyl)-diglucoside-5-glucoside, cyanidin 3-(feruloyl)(sinapoyl)-diglucoside-5-glucoside, and cyanidin 3-(sinapoyl)(sinapoyl)-diglucoside-5-glucoside, respectively). These cyanidin-derivatives all had an acetyl diglucoside and a glucoside group, but the only differences at their acetyl groups were feruloyl and sinapoyl, which made them difficult to separate at normal conditions. However, these compounds could be separated linearly with the temperature increased from 25–45 °C. These results show that the column temperature is a critical factor for HPLC separation of RCAs.

It is proposed that the separation of the RCAs by increasing temperatures on the HPLC column be attributed to their different reductions of the elution time with temperature [20,21,22]. The results showed that the retention times of some RCAs were more sensitive to the column temperature. This phenomenon could be explained from a thermodynamic point [21].

According to Snyder’s theory [21], the relationship between the retention factor and column temperature for a given solute can be described as:

$$\log k_{T}=\log k_{R}-a(1/T_{R}-1/T)$$

**where *k_R_* and *T_R_* are the retention factor and (absolute) temperature for a reference condition (e.g**., 298 K) and *k_T_* and *T* are the retention factor and temperature for the solute at a new temperature, *a* being the energy constant. For a given mobile phase and column condition as in this study, the *a* value of each RCA is fixed. Hence, by plotting log*k_T_* versus (1/*T_R_* − 1/*T*) over the experimental temperature range, the *a* value can be obtained from the slope of the plot [20]. Here, the temperature 25 °C is selected as the reference temperature *T_R_*, and the time at the baseline disturbance (3.0 min) was set as the hold-up time. The retention factor *k_T_* and the *a* values for each RCAs are shown in Table 1.

The value of *a* is negatively proportional to the enthalpy for the transfer of the solute from the mobile phase to the stationary phase [20]. The values of *a* were positive for all RCAs, which means that the adsorption process of RCAs was exothermic. The exothermic retention behavior resulted in a decrease in retention with increasing temperature as observed. However, the retention of some anthocyanins may decrease at a different rate, since the slopes of the van’t Hoff curves (Δ*H*) for these anthocyanins were not equal [22]. Therefore, as the temperature increases, the separation between peaks that co-eluted at a low temperature will be much improved. For example, the *a* was 1451 and 1235 for Peaks 9 and 10, respectively. The larger value of Peak 9 resulted in more reduction degrees of the retention time and consequently separated Peaks 9 and 10. It is obvious that the larger the difference of the *a* values for co-eluted peaks, the more feasible it is to apply the temperature effects to get a good resolution. The difference of the *a* values between Peaks 11 and 13 was larger than that between Peaks 12 and 13, which made Peak 11 more easy to separate from Peak 13, as shown in Figure 1.

### 2.2. HPLC-ESI-MS Analysis and Quantification of RCAs

Based on the optimum HPLC conditions, the components of the RCAs extracts were determined by ESI-MS, as listed in Table 1. The anthocyanins profile of red cabbage had been successfully analyzed by means of HPLC-DAD-MS/MS [3]. According to the results, the derivatives of cyanidin were only found in RCAs [3]. Therefore, all 13 peaks can be identified based on the comparison of their retention time and the mass spectrum with the published data [3]. The tentative identification of chemical structures for each peak is listed in Table 2. The results obtained supported the previous observation that the main structure of anthocyanins in red cabbage was cyanidin-3-diglucoside-5-glucosides, the glycoside chains of which can be nonacylated, monoacylated, and diacylated [3,23]. It can be seen that the monoacylated derivatives of cyanidin were predominate in the RCAs. Anthocyanins with acylation have shown good stabilities to light and heating compared to nonacylated anthocyanins [6]. The purified RCAs contained mainly anthocyanins with acyl chain structures, which are beneficial for their application as a natural colorant in the food industry.

### 2.3. HPLC Method Validation and Quantification

The HPLC method established above was valid using C3G as a standard anthocyanin reference. The linearity, limit of detection (LOD), limit of quantification (LOQ), precision, and recovery were assessed. For linearity tests, the equation of linear regression was *y* = 32.51*x* − 28.36 over the concentration range from 5–60 μg/mL (*y* is the peak area in mAU*s; *x* is the concentration in μg/mL; *R*^2^ = 0.9962). The LOD and LOQ were 0.080 μg/mL and 0.267 μg/mL determined at S/N of three and 10, respectively. The relative standard deviation (RSD) and recovery were tested and calculated to assess the precision of the method. The RSD values of intra-day variations were ± 0.58% for peak areas and ± 0.43% for retention times. The recovery was 98.71 ± 2.53% based on the C3G addition.

**Figure 2:**
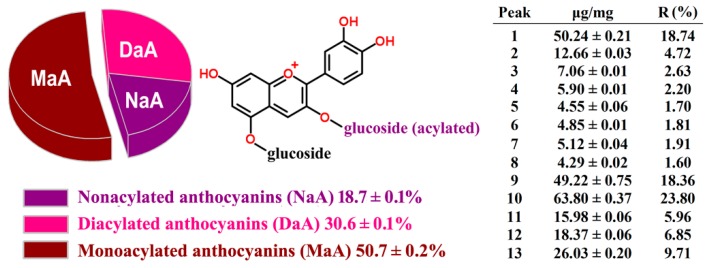
The anthocyanins contents and profiles of the RCA extracts.

The total anthocyanins in the RCAs extracts were determined with 268 ± 2 μg/mg based on the above method. The concentrations of each RCA in the purified powder are listed in Figure 2 along with their relative contents (R%). The composition profiles of nonacylated, monoacylated, and diacylated anthocyanins are also shown in Figure 2. The glycosyl groups of the RCAs were acylated by ferulic acid, sinapic acid, *p*-coumaric acid, and caffeic acid. It can be seen that the derivatives of cyanidin in the extracts were distributed mainly into about 19% nonacylated anthocyanins, 51% monoacylated anthocyanins, and 31% diacylated anthocyanins. Wiczkowski et al. [3] found that nonacylated anthocyanins comprised 27.6% of total RCAs, while monoacylated and diacylated anthocyanins covered 38.4% and 34.1%, respectively. The proportions of each anthocyanin were, however, changed with varietal diversity, maturation time, and cultivation conditions [24,25].

### 2.4. Effect of RCAs against H_2_O_2_-Induced Oxidative Stress in HepG2 Cells

**Figure 3:**
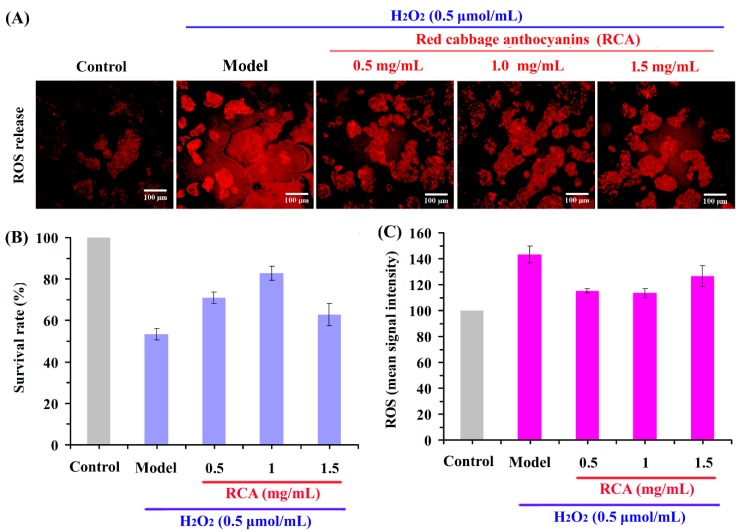
The protective effects of the RCAs against H_2_O_2_-induced HepG2 cell antioxidant damage ((**A**) fluorescence image; (**B**,**C**) fluorescence data analysis).

Because oxidative stress resulted in inevitable damage during metabolism, we investigated whether treatment with the RCAs represses cell death induced by H_2_O_2_. As shown in Figure 3, the cytotoxicity induced by H_2_O_2_ was significantly increased compared to the control group. On the other hand, treatment of the RCAs at all concentrations suppressed the damage triggered by H_2_O_2_. Among three concentrations, the RCAs with 1.0 mg/mL showed more inhibitory effects on oxidative stress-induced cytotoxicity. The result indicated that the RCA extracts could provide the first line of defense to HepG2 cells against oxidative stress. It was supposed that the cells reduced oxidative stress-induced apoptosis by directly scavenging reactive oxygen species (ROS) in the cell.

To clarify the effect of the RCAs on HepG2 cells, the activity levels of ROS in cells were measured. As shown in Figure 3A,C, compared with the control group, the treatment of H_2_O_2_ significantly increase the ROS levels (*p* < 0.05). It is known that H_2_O_2_ can be readily transported through the lipid bilayer of a cell. This initiates the Fenton reaction with metal ions in the cell to form extremely toxic hydroxyl radicals and cause oxidative stress. Results showed that the addition of the RCAs decreased the fluorescence intensity levels of ROS when compared to the positive control sample. When compared to the negative control sample, the values for ROS production increased by about 34%, 19%, 10%, and 20% at the RCA concentrations of 0, 0.5, 1.0, and 1.5 mg/mL, respectively. The addition of all concentrations of the RCAs decreased the ROS values in HepG2 cells. RCAs treated at 0.5, 1.0, and 1.5 mg/mL inhibited about 11%, 18%, and 10% ROS compared to the positive control sample, respectively. RCAs powder produced the largest inhibition rate at the concentration of 1.0 mg/mL powder, which corresponds to 0.21 mg/mL of anthocyanins. The results clearly demonstrated the potential chemoprotective effect of RCAs against oxidative stress induced by H_2_O_2_ on human HepG2 cells.

These results were in line with previous reports that polyphenols could give protection against the increase of intracellular ROS and oxidative damage using different cellular models exposed to different oxidative agents [26,27,28]. The intracellular ROS production was closely related to the state of the antioxidant defense systems of the cell. It has been demonstrated that the protective effects of anthocyanins go beyond their simple antioxidant effect, by activating certain molecular pathways related to the antioxidant response, e.g., increase in antioxidant enzyme activity and the protection of mitochondrial functionality [29,30]. Shih et al. [29] found that anthocyanins could induce the activation of phase II enzymes through the antioxidant response element pathway against H_2_O_2_-induced oxidative stress and apoptosis. Lee et al. [30] recently reported that berry anthocyanins were able to protect the lipopolysaccharide-stimulated RAW 264.7 macrophages against oxidative damage through the activation of the nuclear factor-erythroid 2-related factor 2 (Nrf2). Therefore, any model or the combined actions of an antioxidant mechanism for the RCAs are possible, e.g., by directly scavenging ROS, or by chelating metal ions, thus inhibiting Fenton reactions, or by enhancing the activity of genes involved in the expression of antioxidant enzymes. However, more detailed determinations including enzyme activities are needed. Overall, the study demonstrated that the RCA extracts obtained markedly reduced intracellular ROS production by H_2_O_2_ on HepG2 cells and consequently ameliorated cell apoptosis and improved viability.

## 3. Materials and Methods

### 3.1. Materials

The red cabbage (*Brassica oleracea* var. capitata F. rubra) was grown in Hangzhou, East China. The red cabbage was firstly sealed in a plastic bag and blanched in boiling water for 5 min to inactive enzymes [31]. The blanched red cabbage was instantly cooled and squeezed using a juice extractor with ceramic spiral extrusion (Joyoung JYZ-E3C/E3, Hangzhou, China). The juice was centrifuged to obtain clarified liquid and stored in a refrigerator at −30 °C. The X-5 macroporous resin was obtained from Zhengzhou Qinshi Technology Co., Ltd (Zhengzhou, China).

The human hepatoma HepG2 cell line was obtained from Shanghai Cell Bank (Shanghai, China). Cell culture flasks and black body clear-bottomed 96-well plates (Costar 3631) were purchased from Corning (New York, NY, USA). The MTT Cell Proliferation Assay Kit was purchased from Beyotime Company (Shanghai, China). CellROX Oxidative Stress Reagent and Image-iT™ LIVE Green Caspase Detection Kits were purchased from Molecular Probes, Invitrogen (Eugene, OR, USA).

### 3.2. Purification of RCAs by Resin Adsorption

The adsorption process is shown below [32,33]. The X-5 resin was first pretreated with 95% ethanol, 4% NaOH, and 10% acetic acid to remove organic, alkali, and acid residues according to references. Briefly, 6 bed volumes (BVs) of the juice were pumped into a pretreated resin column at a flow rate of 9 BV per hour for sample loading. Then, the column was eluted with 6 BVs of deionized water to remove the majority of sugars and organic acids. The purple section of RCAs was then eluted by 4 BVs of desorption solution (80% ethanol at pH 4.0) at a flow rate of 9 BV per hour. The eluent fractions were collected and concentrated in a rotary vacuum evaporator at 40 °C to remove ethanol. A dried powder of RCA extracts was obtained by freeze-drying of the concentrated eluent solution. The overall yield was about 110 mg RCAs enriched dry powder per 100 g fresh red cabbage.

### 3.3. HPLC/ESI-MS Characterization

The HPLC/ESI-MS system adopted consisted of an Agilent 1260 system (Agilent Technologies, Wilmington, DE, USA), equipped with a quaternary pump, surveyor plus detector, and ion trap mass spectrometer detector (LCQ Advantage, Thermo, Waltham, MA, USA). Chromatographic separation was performed using an Ultimate LP-C18 column (Ф 4.6 × 250 mm, 5 μm, Yuexu Keji, Shanghai, China). The sample was filtrated through a syringe filter (0.45 μm), and 10 μL of filtrates were injected. The mobile phase consisted of 5% (*v*/*v*) formic acid in water (Solvent A) and methanol (Solvent B) at a flow rate of 1.0 mL/min. The gradient elution program was performed as follows: 0–5 min, 20–30% B; 5–12 min, 30–33% B; 12–18 min, 33–40% B; 18–25 min 40–60% B. The column temperature was maintained using the column oven model, and the detection wavelength was set at 517 nm. The ESI-MS parameters were as follows: positive mode; ESI source voltage, 3.8 kV; capillary voltage, 36 V; sheath gas flow rate, 40 arb; aux gas flow rate, 5 arb; sweep gas flow rate, 0 arb; capillary temperature, 300 °C; and scan range, 50–1500 *m*/*z*.

### 3.4. HPLC Method Validation and Quantification

The HPLC conditions for the method validation and quantification were the same as above. The column temperature was maintained at 45 °C. The linearity of the calibration curve was established by the analysis of the C3G (reference compound) at seven concentrations (5, 10, 20, 30, 40, 50, and 60 μg/mL). The limit of detection (LOD) and limit of quantification (LOQ) were determined at signal-to-noise ratios (S/N) of 3 and 10, respectively [34]. The precision of the developed method was demonstrated by intra-day variations of the sample of 30 μg/mL, which were examined five times within one day. The RSDs for the retention time and peak area were calculated as measures of precision. Recovery was determined by adding C3G to an aqueous solution of the purified RCAs powder.

### 3.5. Cell Culture-Based Assays for Antioxidant Activity

#### 3.5.1. Cell Culture

HepG2 cells were thawed in a 37 °C water bath and maintained in 75-cm^2^ cell culture flasks. Cells were cultured in DMEM medium supplemented with 15% fetal bovine serum, 2 mM l-glutamine, and 1% antibiotics. The cells were kept in a humidified incubator at 37 °C containing atmospheric air and 5% CO_2_. For the subculture, the HepG2 cells were harvested at 70–80% confluence. A trypsin-EDTA solution (0.25% trypsin, 0.02% EDTA) was used to detach cells. The cells that in the logarithmic growth phase were selected and tested.

#### 3.5.2. Cell Count and ROS Assays by HCA

HepG2 cells in the logarithmic growth phase were seeded into 96-well plates at a density of 5000 cells/well in a final volume of 100 μL medium. The cells were allowed to adhere for 12 h. After removing the cell medium, the cells were exposed to 100 μL H_2_O_2_ (500 μM) for 2 h (100 μL culture medium for control). The cell medium was removed again and washed with ice-cold PBS three times. Then, cells were exposed to given concentrations of the RCA solutions prepared with fresh cell medium for 24 h. In our studies, the treatment of HepG2 cells with RCAs at 1000 μg/mL produced no detectable cell toxicity, which was consistent with previous research [35,36].

Following treatments, the viability and ROS levels of the treated cells were measured based on a previous method [19]. Briefly, the cells were loaded with different fluorescent probes including the Hoechst 33342 kit (Sigma, Missouri, USA) and CellROX^®^ Oxidative Stress Reagent. After incubation with fluorescence probes, the plate with cells was scanned from the bottom using an ImageXpress Micro XLS HCS system (Molecular Devices, San Francisco, CA, USA). A DAPI filter cube (exciter (Ex) 377/50, emitter (Em) 447/60; center wavelength (nm)/bandpass width (nm)) was used for Hoechst 33342. A Cy5 filter cube (Ex 628/40, Em 692/40) was used for the ROS dye [19]. The collected images were analyzed with the MetaXpress (Molecular Devices, San Francisco, CA, USA). Experiments were performed in triplicate, and data are shown as the mean ± standard deviation (SD).

## 4. Conclusions

In conclusion, new methods were established for the chemical and cellular assays of the RCA extracts. The effects of column temperature on the HPLC resolution of RCAs were studied and discussed. A good HPLC peak resolution can be achieved at 45 °C and used for the quantification and qualification of the RCAs. The anthocyanins in the extracts were all derivatives of cyanidin (268 ± 2 μg/mg), mainly with 19% nonacylated, 51% monoacylated, and 31% diacylated structures with ferulic, sinapic, *p*-coumaric, and caffeic acids. The RCA extracts markedly reduced intracellular ROS production by H_2_O_2_ on HepG2 cells and consequently ameliorated cell apoptosis and improved viability.

## Figures and Tables

**Table 1:** The retention factor *k_T_* and *a* values for each red cabbage anthocyanin (RCA) ^1^.

Peak No.	*k_25_*	*k_30_*	*k_35_*	*k_40_*	*k_45_*	*a*
1	1.110	1.074	0.954	0.869	0.751	2251
2	1.750	1.703	1.568	1.476	1.329	1552
3	3.426	3.100	2.803	2.542	2.244	2057
4	3.909	3.331	3.013	2.728	2.397	2095
5	4.298	3.591	3.270	2.963	2.609	2036
6	5.334	4.363	4.006	3.633	3.183	2011
7	5.630	4.678	4.307	3.927	3.437	1959
8	6.006	5.289	4.806	4.304	3.730	2231
9	6.737	6.518	6.134	5.712	5.193	1451
10	6.737	6.599	6.299	5.916	5.442	1235
11	7.076	6.890	6.726	6.517	6.286	590
12	7.076	6.952	6.783	6.611	6.453	482
13	7.076	6.952	6.824	6.672	6.488	443

^1^ The retention factor was calculated by *k_T_* = (*t_T_* − *t_0_*)/*t_0_*, with *t_0_* = 3 min.

**Table 2:** Identification of each anthocyanins in the RCA extracts by HPLC-ESI-MS.

Peak	*t_R_*/min	M^+^ *m*/*z*	Tentative Identification
1	6.14	773	Cyanidin-3-diglucoside-5-glucoside
2	8.14	979	Cyanidin-3-(sinapoyl)-diglucoside-5-glucoside
3	11.12	1081	Cyanidin-3-(caffeoyl)(p-coumaroyl)-diglucosides-5-glucoside
4	11.67	1111	Cyanidin-3-(feruloyl)-triglucosides-5-glucoside
5	12.39	1141	Cyanidin-3-(sinapoyl)-triglucoside-5-glucoside
6	14.39	1287	Cyanidin-3-(feruloyl)(feruloyl)-triglucoside-5-glucoside
7	15.29	1317	Cyanidin-3-(feruloyl)(sinapoyl)-triglucoside-5-glucoside
8	16.11	935	Cyanidin-3-(caffeoyl)-diglucoside-5-glucoside
9	19.73	919	Cyanidin-3-(p-coumaroyl)-diglucoside-5-glucoside
10	20.45	949	Cyanidin-3-(feruloyl)-diglucoside-5-glucoside
11	22.88	1125	Cyanidin-3-(feruloyl)(feruloyl)-diglucoside-5-glucoside
12	23.42	1155	Cyanidin-3-(feruloyl)(sinapoyl)-diglucoside-5-glucoside
13	23.78	1185	Cyanidin-3-(sinapoyl)(sinapoyl)-diglucoside-5-glucoside

## References

[1] Dangles O., Fenger J.A. (2018). The chemical reactivity of anthocyanins and its consequences in food science and nutrition. Molecules.

[2] Rose P.M., Cantrill V., Benohoud M., Tidder A., Rayner C.M., Blackburn R.S. (2018). Application of anthocyanins from blackcurrant (*Ribes nigrum* L.) fruit waste as renewable hair dyes. J. Agric. Food Chem..

[3] Wiczkowski W., Szawara-Nowak D., Topolska J. (2013). Red cabbage anthocyanins: Profile, isolation, identification, and antioxidant activity. Food Res. Int..

[4] Wiczkowski W., Szawara-Nowak D., Topolska J. (2015). Changes in the content and composition of anthocyanins in red cabbage and its antioxidant capacity during fermentation storage and stewing. Food Chem..

[5] Moloney M., Robbins R.J., Collins T.M., Kondo T., Yoshida K., Dangles O. (2018). Red cabbage anthocyanins: The influence of D-glucose acylation by hydroxycinnamic acids on their structural transformations in acidic to mildly alkaline conditions and on the resulting color. Dyes Pigments.

[6] Scalzo R.L., Genna A., Branca F., Chedin M., Chassaigne H. (2008). Anthocyanin composition of cauliflower (*Brassica oleracea* L. var. botrytis) and cabbage (*B. oleracea* L. var. capitata) and its stability in relation to thermal treatments. Food Chem..

[7] Zhang J., Wang Z., Liu X. (2016). Characterization of acylated anthocyanins in red cabbage via comprehensive two-dimensional high performance liquid chromatography and HPLC-MS. J. Food Process. Pres..

[8] Willemse C.M., Stander M.A., Tredoux A.G.J., Villiers A.D. (2014). Comprehensive two-dimensional liquid chromatographic analysis of anthocyanins. J. Chromatogr. A.

[9] Buko V., Zavodnik I., Kanuka O., Belonovskaya E., Naruta E., Lukivskaya O., Kirko S., Budryn G., Żyżelewicz D., Oracz J. (2018). Antidiabetic effects and erythrocyte stabilization by red cabbage extract in streptozotocin-treated rats. Food Funct..

[10] Degenhardt A., Knapp H., Winterhalter P. (2000). Separation and purification of anthocyanins by high-speed countercurrent chromatography and screening for antioxidant activity. J. Agric. Food Chem..

[11] Nordberg J., Arnér E.S. (2001). Reactive oxygen species, antioxidants, and the mammalian thioredoxin system. Free Radicals Biol. Med..

[12] Chaudhary A., Bag S., Banerjee P., Chatterjee J. (2017). Honey extracted polyphenolics reduce experimental hypoxia in human keratinocytes culture. J. Agric. Food. Chem..

[13] Li F., Zhang X., Zheng S., Lu K., Zhao G., Ming J. (2016). The composition.; antioxidant and antiproliferative capacities of phenolic compounds extracted from tartary buckwheat bran [*Fagopyrum tartaricum*, (L.) Gaerth]. J. Funct. Foods.

[14] Omar S.H., Kerr P.G., Scott C.J., Hamlin A.S., Obied H.K. (2017). Olive (*Olea europaea* L.) Biophenols: A Nutriceutical against Oxidative Stress in SH-SY5Y Cells. Molecules.

[15] Guo Y., Sun L., Yu B., Qi J. (2017). An integrated antioxidant activity fingerprint for commercial teas based on their capacities to scavenge reactive oxygen species. Food Chem..

[16] Salla S., Sunkara R., Ogutu S., Walker L.T., Verghese M. (2016). Antioxidant activity of papaya seed extracts against H_2_O_2_ induced oxidative stress in HepG2 cells. LWT-Food Sci. Technol..

[17] Sankhari J.M., Thounaojam M.C., Jadeja R.N., Devkar R.V., Ramachandran A.V. (2012). Anthocyanin-rich red cabbage (*Brassica oleracea* L.) extract attenuates cardiac and hepatic oxidative stress in rats fed an atherogenic diet. J. Sci. Food Agr..

[18] Saluk J., Bijak M., Posmyk M.M., Zbikowska H.M. (2015). Red cabbage anthocyanins as inhibitors of lipopolysaccharide-induced oxidative stress in blood platelets. Int. J. Biol. Macromol..

[19] Qu D., Gu Y., Feng L., Han J. (2017). High content analysis technology for evaluating the joint toxicity of sunset yellow and sodium sulfite in vitro. Food Chem..

[20] Dolan J.W. (2002). Temperature selectivity in reversed-phase high performance liquid chromatography. J. Chromatogr. A.

[21] Gant J.R., Dolan J.W., Snyder L.R. (1979). Systematic approach to optimizing resolution in reversed-phase liquid chromatography, with emphasis on the role of temperature. J. Chromatogr. A.

[22] Khalaf R., Baur D., Pfister D. (2015). Optimization of reversed-phase chromatography methods for peptide analytics. J. Chromatogr. A.

[23] Prior R.L., Wu X., Schaich K. (2005). Standardized methods for the determination of antioxidant capacity and phenolics in foods and dietary supplements. J. Agric. Food Chem..

[24] Ahmadiani N., Robbins R.J., Collins T.M., Giusti M.M. (2014). Anthocyanins contents, profiles, and color characteristics of red cabbage extracts from different cultivars and maturity stages. J. Agric. Food Chem..

[25] Wiczkowski W., Piskuła M.K. (2004). Food flavonoids. Pol. J. Food Nutr. Sci..

[26] Yeh C.T., Ching L.C., Yen G.C. (2009). Inducing gene expression of cardiac antioxidant enzymes by dietary phenolic acids in rats. J. Nutr. Biochem..

[27] Yang G., Liao J., Kim K., Yurkow E.J., Yang C. (1998). Inhibition of growth and induction of apoptosis in human cancer cell lines by tea polyphenols. Carcinogenesis.

[28] Huang W.Y., Wu H., Li D.J., Song J.F., Xiao Y.D., Liu C.Q., Zhou J.Z., Sui Z.Q. (2018). Protective effects of blueberry anthocyanins against H_2_O_2_-induced oxidative injuries in human retinal pigment epithelial cells. J. Agric. Food Chem..

[29] Shih P.H., Yeh C.T., Yen G.C. (2007). Anthocyanins induce the activation of phase II enzymes through the antioxidant response element pathway against oxidative stress-induced apoptosis. J. Agric. Food Chem..

[30] Lee S.G., Kim B., Yang Y., Pham T.X., Park Y.K., Manatou J., Koo S.I., Chun O.K., Lee J.Y. (2014). Berry anthocyanins suppress the expression and secretion of proinflammatory mediators in macrophages by inhibiting nuclear translocation of NF-kB independent of NRF2-mediated mechanism. J. Nutr. Biochem..

[31] Cheng L., Fang S., Ruan M. (2015). Influence of blanching pretreatment on the drying characteristics of cherry tomato and mathematical modeling. Int. J. Food Eng..

[32] Chen Y., Zhang W., Zhao T., Li F., Zhang M., Li J., Zou Y., Wang W., Cobbina S.J., Wu X. (2016). Adsorption properties of macroporous adsorbent resins for separation of anthocyanins from mulberry. Food Chem..

[33] Buran T.J., Sandhu A.K., Li Z., Rock C.R., Yang W., Gu L. (2014). Adsorption/desorption characteristics and separation of anthocyanins and polyphenols from blueberries using macroporous adsorbent resins. J. Food Eng..

[34] Liang X., Wu H., Su W. (2014). A rapid UPLC-PAD fingerprint analysis of chrysanthemum morifolium ramat combined with chemometrics methods. Food Anal. Method..

[35] Yan F., Chen Y., Azat R., Zheng X. (2017). Mulberry Anthocyanin Extract Ameliorates Oxidative Damage in HepG2 Cells and Prolongs the Lifespan of Caenorhabditis elegans through MAPK and Nrf2 Pathways. Oxid. Med. Cell. Longev..

[36] Hwang Y.P., Choi J.H., Choi J.M., Chung Y.C., Jeong H.G. (2011). Protective mechanisms of anthocyanins from purple sweet potato against tert-butyl hydroperoxide-induced hepatotoxicity. Food Chem. Toxicol..

